# Proprioceptive Flexible Fluidic Actuators Using Conductive Working Fluids

**DOI:** 10.1089/soro.2017.0012

**Published:** 2018-04-01

**Authors:** Tim Helps, Jonathan Rossiter

**Affiliations:** ^1^ Department of Engineering Mathematics, University of Bristol, Bristol, United Kingdom.; ^2^ Bristol Robotics Laboratory, Bristol, United Kingdom.

**Keywords:** soft, proprioception, flexible, fluidic, actuator, sensor

## Abstract

Soft robotic systems generally require both soft actuators and soft sensors to perform complex functions. Separate actuators and sensors are often combined into one composite device when proprioception (self-sensing) is required. In this article, we introduce the concept of using a conductive liquid to perform both the sensing and actuation functions of a proprioceptive soft actuator. The working fluid drives actuator deformation while simultaneously acting as a strain-sensing component for detecting actuator deformation. The concept is examined and demonstrated in two proprioceptive flexible fluidic actuators (FFAs) that use conductive liquids as their working fluids: a linear actuator and a bending actuator. In both cases, we show that resistance can be used to infer strain. Some hysteresis and nonlinearity are present, but repeatability is high. The bandwidth of resistance as a sensing variable in the bending FFA is tested and found to be ∼3.665 Hz. Resistance is demonstrated as a feedback variable in a control loop, and the proprioceptive bending FFA is controlled to respond to step input and sinusoidal target functions. The effect of temperature on resistance–strain behavior is also examined, and we demonstrate how measurement of volume and resistance can be used to detect when the actuator is constrained. Biocompatible proprioceptive soft actuators such as those presented in this article are ideal for use in low-cost bionic healthcare components such as orthotics, prosthetics, or even replacement muscles.

## Introduction

Soft robotics is a rapidly emerging field whose focus is to develop lower stiffness alternatives to the hard and rigid systems of conventional robotics.^1–3^ Soft robotic systems typically exploit soft actuators that have high compliances compared with their hard counterparts. Soft pneumatic and hydraulic actuators (also called flexible fluidic actuators or FFAs) are particularly popular in this respect because of their simplicity, inherent low stiffness, and backdrivability.^4,5^ Historically, the most common FFA in research has been the McKibben artificial muscle actuator,^6^ although many alternative pneumatic structures have been studied for decades.^7^ More recently, more complex pneumatic structures have been introduced, such as pleated pneumatic artificial muscles,^8^ asymmetrically stiff structures that bend when inflated,^9–11^ computationally designed inflating pouch actuators,^12^ and vacuum driven buckling tensile actuators.^13^ Bending actuators that contain pneumatic networks (PneuNets) have become increasingly popular.^14,15^ Recently, complex FFAs have also been actuated hydraulically, with liquid as the working fluid rather than air.^16^

Soft robotic systems require sensors with compliances as high as those of their soft actuators. In particular, strain and shape sensing are often necessary for systems whose functionality requires them to alter their form. Various soft strain sensors have been proposed, including permanent magnetic elements encased in soft materials,^17^ capacitive sensors using dielectric elastomers,^18^ and systems where strain is detected optically as a result of wrinkling of thin films,^19^ or through changes in resistance due to the formation of parallel microcracks.^20^ Soft structures that contain conductive fluid, through which electrical properties are altered in response to changes in the shape of the sensor, have been studied in detail.^21–27^ Typically, the conductive fluid used is eutectic gallium–indium (EGaIn liquid metal) because it is liquid at room temperature and has lower toxicity and reactivity compared with mercury (Hg). However, alternative conductive fluids have also been used, particularly ionic liquids^28–30^ and ionic solutions such as aqueous sodium chloride (NaCl).^31,32^ Eutectic gallium–indium and ionic liquids have also been combined in a single soft strain sensor^33^ and used separately in soft pressure sensors.^34–36^

**FIG. 1.**
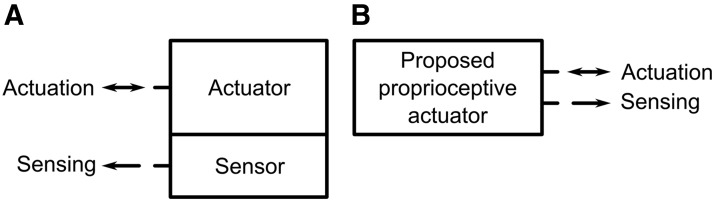
Block diagram comparison between traditional actuator-sensor system and proposed proprioceptive actuator. **(A)** Traditional arrangement when feedback is necessary: a standalone actuator combined with a standalone sensor. **(B)** The proposed proprioceptive FFA concept through which the same working fluid performs both actuation and sensing tasks, reducing complexity and actuator-sensor system volume and mass. FFA, flexible fluidic actuator.

FFAs and soft sensors are often combined when self-sensing is required (Fig. 1A). Examples of self-sensing FFAs include a modular elastomer orthotic sleeve that includes pneumatic McKibben actuators and liquid metal sensors,^37^ integration of strain and pressure sensors into a pneumatic bending actuator,^38^ and the proposal of the inclusion of ionic liquid sensors alongside a pneumatically actuated, variable stiffness manipulator for medical applications.^32^ Most recently, stretchable optical waveguides have been demonstrated as strain sensors built into FFAs in a soft prosthetic hand.^39^ Alternatively, measurement of fluid pressure inside FFAs for robotic surgery has been used to detect force^40^ or infer strain.^41,42^

One advantage of soft actuators over their rigid counterparts is that their deformable structure allows some useful tasks, such as grasping, to be performed without the requirement of sensors.^14,43^ However, systems performing more complex tasks, especially those where feedback is required (such as position control in robotic surgery, touch-sensing in prosthetics, and for operation in changing environments), will benefit greatly from proprioceptive soft actuators.

In this article, we introduce the concept of using a conductive liquid to perform both of the essential roles of a proprioceptive soft actuator: (1) as the working fluid to drive actuator deformation and (2) as the strain-sensing component to detect actuator deformation (Fig. 1B). This can greatly reduce the complexity and cost of the actuator sensor system. In fact, the actuator volume footprint and mass are practically unchanged by the addition of sensing capabilities (only electrodes must be added, which are very small and lightweight). As such, using this concept, existing systems using FFAs can be easily modified to make those actuators proprioceptive without alteration of the overall system design.

To create a proprioceptive soft actuator, we select an existing FFA or design a new FFA form. The actuator is modified by the addition of electrodes at each end of the section to be sensed and filled with a conductive working fluid (aqueous sodium chloride and later tap water are used in this article). By altering the volume or pressure of the working fluid (using a pump or syringe), the actuator deforms, which also alters the resistance of the conductive working fluid. This resistance change is measured using the electrodes and used to infer actuator deformation.

## Materials and Methods

We will first consider how resistance measurement in conductive fluids may be achieved in soft actuator systems. The resistance of any material (solid or liquid) varies according to the dimensions of that material. Pouillet's law describes the resistance of an ideal single material conductor with a uniform cross section as a function of its resistivity and dimensions:

\begin{align*}
R = \rho \frac { l }  { A } , \tag { 1 } 
\end{align*}

where *R* is resistance, $\rho$ is resistivity, *l* is length, and *A* is cross-sectional area. For typical materials with a positive Poisson's ratio, application of tensile stress will cause an increase in length and a reduction in cross-sectional area, increasing resistance. Resistance in liquids may be described using the same law, although the shape of the liquid will depend on the shape of the container. In previous conductive liquid sensors, the liquid is sealed within a container, and thus (assuming the liquid is incompressible), volume is conserved. As such, when stress is applied to the sensor, length increases while cross-sectional area decreases.

**FIG. 2.**
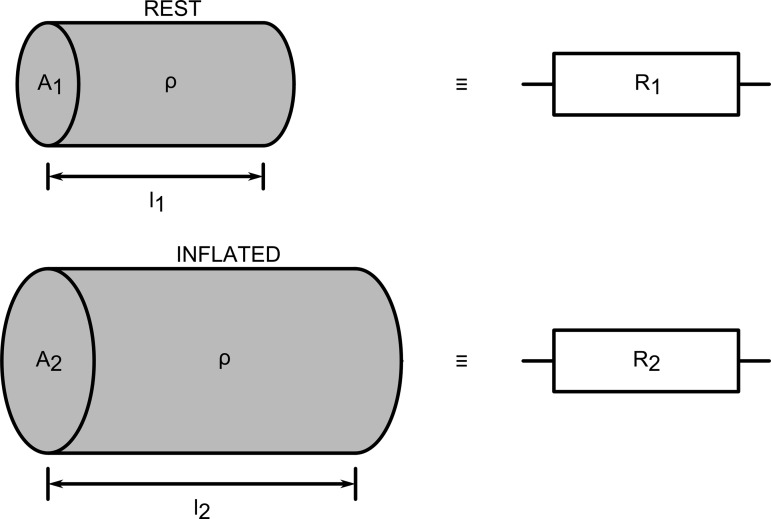
The mechanism of resistance change in a proprioceptive FFA. As the actuator is inflated, variation in the length and cross-sectional area of the conductive volume causes variation in resistance.

In the experiments described in this article, fluid volume is not conserved. However, Pouillet's law can still be used to describe the change in resistance of the conductive liquid. The actuators are inflated or deflated, which, depending on the geometry of the actuator, may increase or decrease the end-to-end resistance of the liquid contained within (Fig. 2). Note that while the terms “inflate” and “deflate” are typically reserved for the filling and emptying of an object with air or gas, we use them in this article to mean fill or empty with any fluid, for the sake of convenience. In the experiments described in this article, aqueous sodium chloride (saltwater) was initially used as the conductive liquid, because of its low cost, nontoxicity, and ready availability.

Conductance in saltwater is a result of the movement of sodium and chloride ions, which migrate toward the cathode and anode, respectively. Consequently, the conductance under direct current (DC) is initially high but reduces as the ions congregate near their respective electrodes and fewer charge carriers are available. To maintain the conductance of the saltwater solution, we used a high-frequency (typically 1 kHz) alternating current (AC) signal. At such high frequencies, the ions oscillate within the solution rather than migrating in one direction because the direction of the Lorentz force acting on them is continuously reversing. If the potential difference across the solution is greater than the relevant oxidation and reduction potentials, electrolysis occurs and chlorine, hydrogen, and sodium hydroxide are produced. To avoid this, the voltage across the saltwater solution should be kept low. The conductance of saltwater is known to vary with temperature, so all experiments were performed in a temperature-controlled room at 20°C.

Since an AC signal was used, the relationship between voltage and current for this system is governed by the system's electrical impedance. In this work, because the actuator dynamics is much slower than the electrical dynamics, we use root mean square (RMS) values of voltage and current. As such, the quantity of interest is only the magnitude of complex impedance and is equivalent to DC resistance.

To determine the resistance of a volume of saltwater, we used a galvanostat (HA-151B; Hokuto Denko, Japan), which maintained a high-frequency AC sine wave current flow within the saltwater solution. The maximum current setting error was ±1% and maximum voltage recording error ±0.1%. The voltage required to achieve this current flow was recorded. Control signals were generated and data were captured using a data acquisition device (NI USB-6229 BNC; National Instruments). The data acquisition sample rate was 10 kHz. To calculate resistance, for each sine wave period, the RMS value of the alternating component of the voltage across the saltwater was divided by the RMS value of the current enforced, according to Ohm's law.

In later experiments, once demonstrated to be suitable for soft actuator proprioception, tap water (which contains trace salts, making it slightly conductive) was used in the place of saltwater. Tap water was obtained on-site at Bristol Robotics Laboratory in July and August 2017 (Bristol Water, Filton and Northville water supply zone). The conductivity of local tap water reported by Bristol Water at the time of writing was 591 μS/cm.^44^

## Characterization

To provide a baseline for analysis, the resistance–length relationship of a column of saltwater, made by mixing a known quantity of sodium chloride (NaCl) with water, was determined using a simple experimental set up. A 50-mm-diameter graduated cylinder was filled with 0.5 L of saltwater (ratio of salt to water 1:1000 by mass). A pair of circular electrodes were constructed, which consisted of copper tape attached to two 47-mm-diameter laser cut acrylic cylinders. The diameter of the electrodes was chosen to be as close as possible to the diameter of the graduated cylinder to match the geometry of an ideal conductor system, however, with diameters greater than 47 mm the electrodes tended to jam. The acrylic cylinders had central holes so that wire connections could be soldered to the rear of the copper tape, maintaining a smooth surface in contact with the saltwater for conduction. On both electrodes, small 5-mm-diameter semicircular cutouts were included at four equally spaced locations around the perimeter of the electrode to allow the flow of saltwater past the electrode when moved. The lower electrode was placed at the bottom of the graduated cylinder, and the upper was attached to a thread, which was routed through a pair of pulleys to a moving linear stage. The linear stage was moved by a linear actuator (LACT8P-12V-20; Pololu) and its displacement was recorded using a laser displacement meter (LK-G502; Keyence, Japan).

**FIG. 3.**
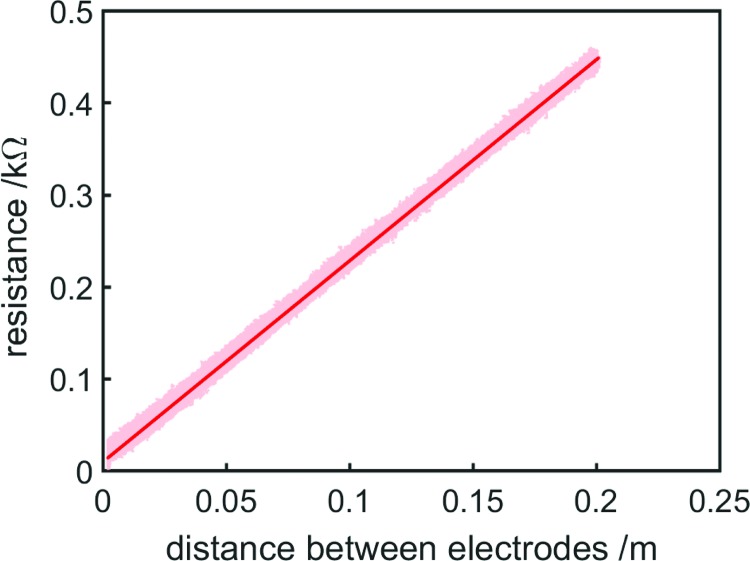
Resistance variation with length for a 50-mm-diameter cylinder of saltwater (ratio of salt to water 1:1000), collected while moving the electrode from maximum to minimum length and back three times. Data where the electrodes were nearer to one another than 0.002 m have not been plotted because near this point at times the electrodes touched one another, causing nonlinearities in resistance. A first-order line of best fit (least-squares) is shown in *solid red*, with experimental data shown as a *light red* point cloud. Color images available online at www.liebertpub.com/soro

In this experiment, the amplitude of the current sine wave was 1 mA and its frequency was 1000 Hz. The upper electrode was moved from a distance of 0.2 m until it touched the lower electrode, and back, three times over the course of 120 s. Figure 3 shows the variation of resistance with length for the column of saltwater. Resistance variation with length for the cylinder of saltwater was linear and proportional to length, as was expected from Pouillet's Law, since resistivity and cross-sectional area were invariant.

This experimental setup, in which the electrodes have the same cross-sectional area as the column of conducting fluid, is suitable for determining ideal resistance–length relationships without the influence of effects such as fringing (which will be discussed later) but impractical for real-world actuators. The electrodes limit the flow of liquid past them in a similar manner to that of a dashpot, inhibiting high-speed movement, and any real soft robotic actuator will inevitably have a variable cross-sectional area.

**FIG. 4.**
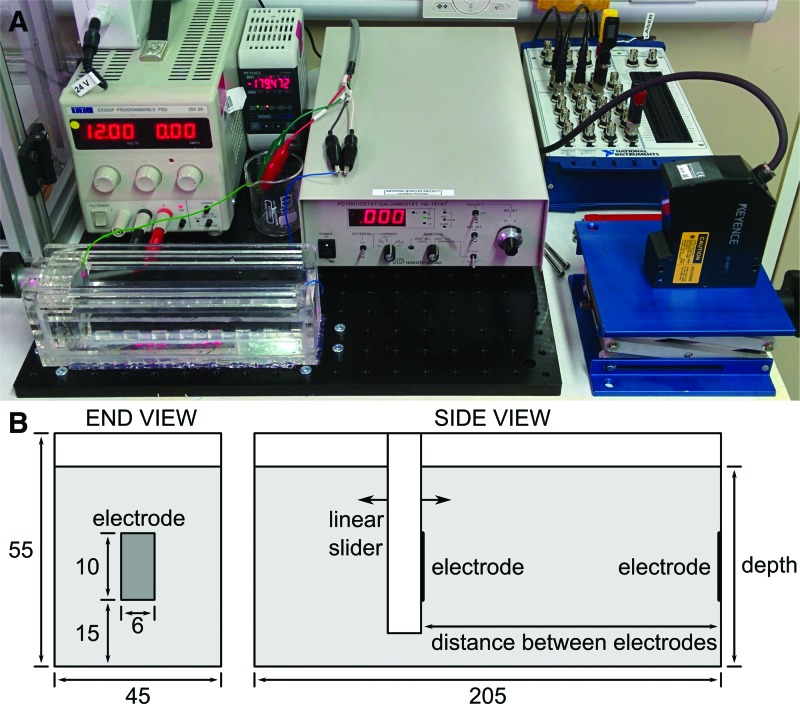
**(A)** Experimental setup used for initial experiments. Clockwise from center: galvanostat, data acquisition module, laser displacement meter, conductive fluid bath and movable electrode, linear actuator power supply, laser displacement meter display. **(B)** Detailed drawing of bath and electrodes with dimensions in mm. Color images available online at www.liebertpub.com/soro

For analysis that is more applicable to soft robotic actuators, we performed experiments with electrodes considerably smaller than the cross-sectional area of the conductive liquid. The experimental setup for these experiments is shown in Figure 4. The graduated cylinder was replaced with a rectangular bath with interior dimensions 205 by 55 by 45 mm. Two electrodes made of copper tape of dimensions 6 by 10 mm were mounted at either end of the bath, with the left electrode attached to a linear slider, which could be moved by hand or by the linear actuator. The laser displacement meter was used to record displacement.

This setup was used to investigate the effect of salinity (salt to water ratios from 1:10 to 1:10,000), current sine wave frequency (1 to 1000 Hz), and saltwater depth (30 to 50 mm). For experiments in which depth was not varied, it was held constant at 50 mm. For some experiments, the linear actuator was disconnected and the left electrode was moved manually.

**FIG. 5.**
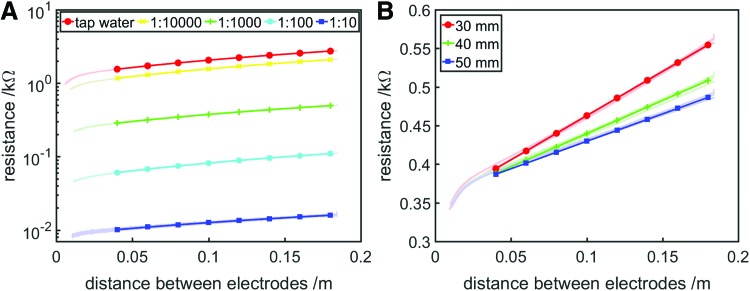
Resistance variation with interelectrode distance for a rectangular bath of saltwater. **(A)** Resistance–distance behavior as salinity (as a ratio of salt to water) is varied. At each salinity, the electrodes were moved from minimum to maximum distance and back once by the linear actuator. The data are plotted on a logarithmic *y*-axis to capture the full range of resistance-length variation. **(B)** Resistance-distance behavior as saltwater depth is varied. At each depth, the electrodes were moved from minimum to maximum distance and back twice by the linear actuator. In both cases, the relationships are linear above ∼0.04 m. First-order least-squares best fit lines (using data from 0.04 m and above) are shown as *solid lines* with markets added to aid with identification, and experimental data are shown as *lightly colored* point clouds. Color images available online at www.liebertpub.com/soro

Figure 5A shows resistance variation with interelectrode distance for saltwater solutions of varying salinities, calculated using a 0.5 mA 1000 Hz current sine wave. In these experiments, the linear slider was moved from minimum to maximum length and back by the linear actuator. Resistance variation with interelectrode distance for tap water was also investigated and resulted in similar behavior (although at a higher resistance). Above 0.04 m, resistance varied linearly with interelectrode distance. The gradients of the first-order best fit lines are 8.461, 6.616, 1.483, 0.352, and 0.0413 kΩ/m, respectively. As expected, increasing the salt content of the solution increased its conductivity.

We determined in the initial saltwater cylinder experiment (Fig. 3) that resistance variation with length in a column of saltwater behaves according to Pouillet's Law. To determine whether saltwater resistance variation with area can also be described by theory, we investigated the influence of saltwater depth on resistance–length behavior. Figure 5B shows resistance variation with interelectrode distance calculated using a 1 mA 1000 Hz current sine wave, with varying depths of 1:1000 salinity solution, with the linear slider moved from minimum to maximum distance and back twice by the linear actuator. The gradients of the resistance–length relationship at distances greater than 0.04 m for each depth are 1.144, 0.8586, and 0.7103 kΩ/m, respectively; gradient thus being inversely proportional to the depth of saltwater, confirming the validity of Pouillet's Law for modeling variation in area for these systems.

**FIG. 6.**
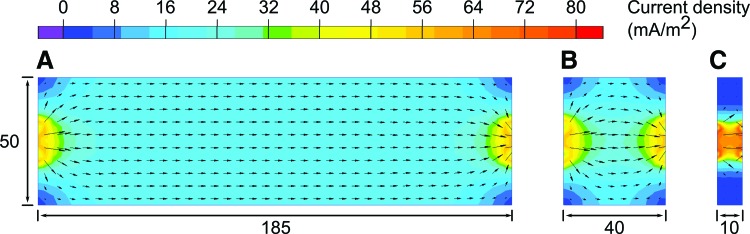
Conduction simulation of three 50-mm-deep saltwater baths, with 10-mm-tall small electrodes at each end. Bath lengths are **(A)** 185 mm, **(B)** 40 mm, and **(C)** 10 mm. The legend shows current density in units of mA/m^2^. The *arrows* are vectors showing the magnitude and direction of current flow at 5 mm intervals. Color images available online at www.liebertpub.com/soro

Results from this experiment differed from those for the ideal case, in that as the interelectrode distance became low, the relationship between resistance and length became nonlinear. This can be attributed to boundary effects; the cross-sectional area of the electrodes was much smaller than the cross-sectional area of the saltwater itself. As such, near the electrodes, electric current flowed radially. This effect is shown qualitatively in Figure 6, a conduction simulation of the bath using Field-Modeling software (QuickField; Tera Analysis Ltd., Denmark).^45^ In this simulation, the current flow was 1 mA.

In Figure 6A and B, current flow is characterized by radial current flow at each end of the bath (near the electrodes) and horizontal current flow in the central region. One way of considering these systems is as three resistive volumes in series. The radial current flow at each end in Figure 6A is very similar to that of Figure 6B, so these resistive volumes can be considered to have very similar resistances. The central region is shorter in Figure 6B compared with Figure 6A, but since current flow is almost uniformly horizontal, the region is described using Pouillet's Law, and the change in resistance as bath length reduces from 0.185 to 0.04 m is linear, as it is in Figure 5.

As the bath length is further reduced, the central region disappears entirely (Fig. 6C), and end regions of the bath begin to interact with each other, altering current flow behavior and resulting in the nonlinear resistance–length relationship observed. Additional effects occurring near to the electrodes, such as induced electrical double layers, may also contribute to the observed nonlinearity but are beyond the scope of this article.

**FIG. 7.**
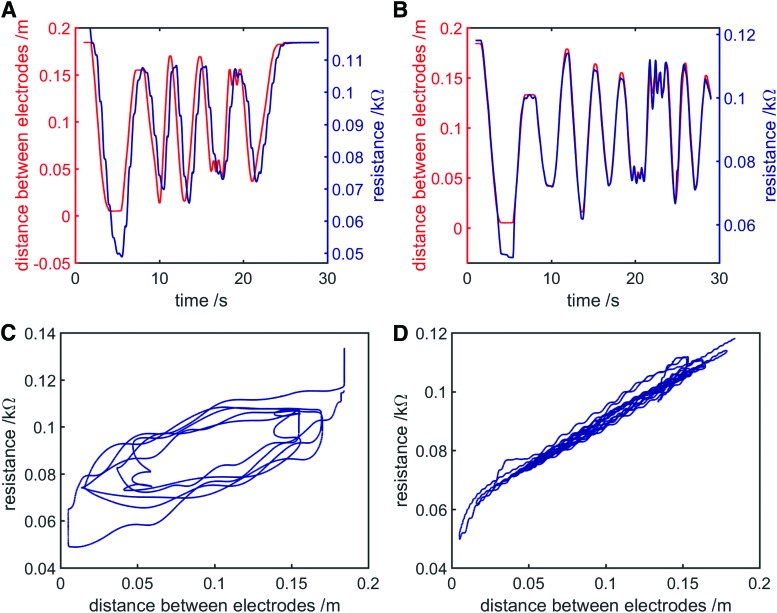
Assessing saltwater resistance as a sensing method—the left electrode was manually moved at varying speeds over a 30-s test period and resistance and interelectrode distance were recorded. **(A)** Distance and resistance with respect to time, current sine wave frequency 1 Hz. **(B)** Distance and resistance with respect to time, current sine wave frequency 10 Hz. **(C)** Resistance variation with distance, current sine wave frequency 1 Hz. **(D)** Resistance variation with distance, current sine wave frequency 10 Hz. Color images available online at www.liebertpub.com/soro

Characterization results showing resistance variation with length, salinity, and depth for volumes of saltwater have suggested that conductive working fluids might be useful as a variable resistive element in a fluidic actuator to infer strain. However, still to be determined was the suitable frequency of the excitation signal. Figure 7 shows variation in both resistance and interelectrode gap with respect to time, as interelectrode gap within the saltwater bath was manually varied at a range of speeds over the course of a 30-s test period. Current sine wave amplitude was 50 mA and the ratio of salt to water was 1:100. For these tests, current sine wave frequency was varied, to test how well resistance could predict length at lower frequencies.

At 10 Hz (Fig. 7B, D) and above, resistance was a good predictor of length. At 1 Hz (Fig. 7A, C), the relationship between resistance and length was poor. This is because, when excitation frequency is low, resistance variation with time becomes significant. Recall that when DC voltage is applied to an ionic solution, conductance decays as charge carriers migrate toward their respective electrodes, reducing the number of available charge carriers. At 10 Hz, the excitation signal polarity reverses frequently enough that the amplitude of this conductance decay is small (a large number of available charge carriers always remain in the solution), and the total resistance variation of the saltwater bath is mostly due to changes in length. At 1 Hz, the excitation signal polarity reverses far less frequently, and the amplitude of conductance decay is large (many charge carriers have migrated to their attracting electrodes before polarity is reversed). As such, total resistance variation is a combination of resistance variation due to change in bath length and conductance decay due to a reduction of available charge carriers. Thus, resistance is a poor predictor of distance between electrodes at low excitation signal frequencies. When using an ionic conductive fluid as a predictor of length, this problem can be avoided by using a sufficiently high excitation frequency.

## Proprioceptive FFAs

**FIG. 8.**
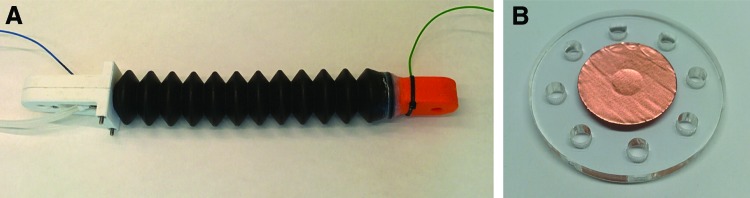
**(A)** A proprioceptive linear FFA. **(B)** Electrode structures contained within the proprioceptive linear FFA, which were composed of acrylic discs with holes for fluid flow, and central copper tape electrodes. Color images available online at www.liebertpub.com/soro

A proprioceptive linear FFA was fabricated to test the suitability of the conductive fluid proprioception concept for real-world soft robotic systems. A commercially available rubber bellow (190 mm long, 38 mm maximum diameter) was adapted as a linear actuator (Fig. 8A). The electrode structures consisted of acrylic discs with eight outer 3-mm-diameter holes that allowed the throughflow of conductive liquid (Fig. 8B). A central 16-mm-diameter circle of copper tape formed the electrode proper and as before, wire connections were made at the rear of the electrode through a 5-mm hole in the acrylic disc to maintain a smooth conductive surface. One such electrode structure was inserted into each end of the actuator, aligned so that the electrodes faced one another. Three-dimensional printed adaptors, which fitted inside the ends of the bellow, were fabricated from polylactic acid and sealed using silicone sealant. Wires connected to the electrodes terminated at each end of the actuator, and a barbed Y connector at one end split the outlet between electrode wire and fluid port.

The proprioceptive linear FFA was tested in a simple vertical test rig consisting of an upper mounting point and vertically movable lower slider. The laser displacement meter was used to record the length of the actuator, and resistance was calculated as described previously using the galvanostat. The current sine wave amplitude was 1 mA and frequency 1000 Hz. Care was taken when filling the actuator that no air bubbles remained before testing, because such bubbles would cause incorrect resistance measurements if they covered an electrode due to the low conductivity of air. In a first experiment, 1:1000 saltwater was pumped into and out of the actuator using a gear pump (ZC-A210; Shenzhen Shanhai Technology Ltd., China). In a second experiment, tap water was used in the place of saltwater, and volume was controlled using a syringe driver consisting of a plastic syringe and computer-controlled linear stage (X-LSQ150B-E01; Zaber Technologies, Inc., Canada). The actuator was inflated and then deflated three times during the experiment.

**FIG. 9.**
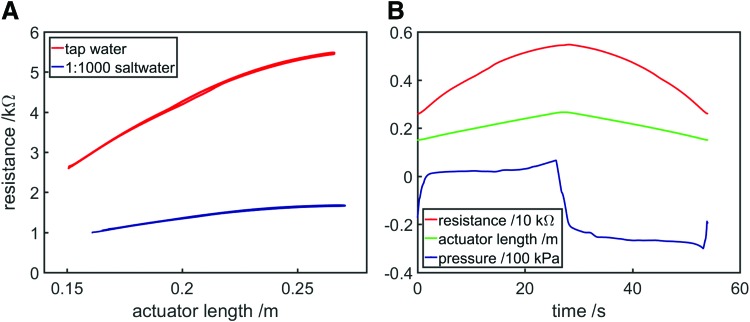
Characterization of the linear proprioceptive FFA, recorded by inflating and deflating the actuator three times automatically. **(A)** Resistance variation with actuator length for 1:1000 saltwater and tap water. Some hysteresis is visible (resistance was slightly lower when the actuator was lengthening compared with when shortening). **(B)** Resistance, actuator length, and pressure variation with time during the first cycle of the tap water experiment. Pressure was considerably lower when the actuator was deflating compared with when the actuator was inflating. Color images available online at www.liebertpub.com/soro

The proprioceptive linear FFA confirmed that resistance could be a good predictor of length for a soft actuator, with resistance monotonically increasing with length (Fig. 9A). A video of the tap water experiment alongside live data is available in Supplementary Video S1 (Supplementary Data are available online at www.liebertpub.com/soro). Video synchronization was performed using a visible LED, which was controlled by the same data acquisition module responsible for inflation and deflation of the actuator and collection of data. Fluid pressure was recorded using a differential pressure sensor (HSCSAAN015PDAA5; Honeywell, NJ). A small amount of hysteresis is visible in Figure 9 and the Supplementary Video S1, with resistance slightly lower when the actuator was lengthening and higher when shortening. Also of note is the highly asymmetrical pressure variation—pressure is considerably lower during deflation compared with inflation (Fig. 9B). This contrasts with the behavior of a typical “balloon-like” system, through which pressure gradually increases during inflation and gradually decreases during inflation. The asymmetry in this case is due to the anisotropic stiffness of the bellows—the axial stiffness is comparatively low and as such the elastic restoring force is low, and therefore, the system behaves more like a syringe through which a positive pressure is required for filling and a negative pressure is required for emptying.

The hysteresis is attributed to the flexibility of the actuator walls. When being inflated, the fluid within the actuator was at a higher pressure than when being deflated. This pressure difference alone cannot explain the disparity in resistance—it has been shown that for a 0.5 M NaCL solution specific conductance varies by less than 5% over a pressure range of 500 bar.^46^ However, the difference in pressure resulted in bulging or buckling of the actuator walls—becoming more convex when the actuator was inflated and concave when deflated. As has been shown in previous experiments (Fig. 5B), an increase in the cross-sectional area of a conductor causes a reduction in resistance, according to Pouillet's Law. When the actuator was inflating (high pressure, bulging walls), the cross-sectional area was increased and thus resistance was reduced, whereas when the actuator was deflating (low pressure, buckling walls), the cross-sectional area was reduced and thus resistance was increased. Although this slight hysteresis was present, repeatability was good. Figure 9 shows results from three expansions and three contractions; the curves are so near to one another that they appear as a single loop.

In contrast to the saltwater bath, where nonlinear behavior occurred when the distance between electrodes was small, here even at low length values, the resistance–length relationship was linear. This is attributed to the fact that even when the linear FFA was fully contracted, it remained greater than 0.15 m long, and the electrodes were still sufficiently far from one another for the behavior to be approximated to that of an ideal conductor. However, when the actuator was near to fully extended, the relationship then started to become nonlinear, that is, resistance increased less than would be expected from a linear relationship. This is also due to the wall flexibility discussed previously. As the actuator was overfilled with saltwater, the amount of bulging of the walls increased, resulting in an even higher cross-sectional area and a further reduction in resistance. This shows how the deformation of proprioceptive FFAs can alter their resistance in a nonlinear manner, which will need to be accounted for when inferring changes in strain or shape.

**FIG. 10.**
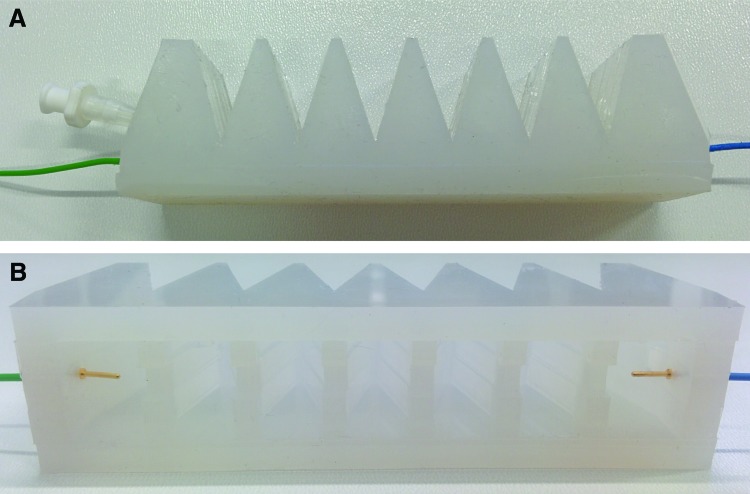
**(A)** A proprioceptive bending FFA. **(B)** The proprioceptive bending FFA part way through fabrication, showing the electrodes neces sary to measure the resistance of the conductive fluid. Color images available online at www.liebertpub.com/soro

Finally, a proprioceptive bending FFA was fabricated, as shown in Figure 10A. The proprioceptive bending FFA most closely resembles the commonly used PneuNet bending actuators.^14,15^ The bending actuator was fabricated using a conventional two-part casting process: first the main structure of the actuator was cast from silicone rubber (Dragon Skin^®^ 30; Smooth-On, Inc.) and then an inextensible mesh layer was added to the base of the actuator, and the structure was sealed with additional silicone rubber. An .stl file of the actuator main structure is available in the Supplementary Data. The only necessary alteration of this process to make the actuator proprioceptive was the addition of electrodes at each end of the actuator. In this case, gold-plated 1-mm-diameter test plugs were used (22.2070-21 22.1007; Multi Contact, Switzerland). These were pushed into the two end walls, protruding into the inner chamber by 7 mm (Fig. 10B), and sealed with silicone adhesive (SilPoxy; Smooth-On, Inc.).

The resistance of the proprioceptive bending FFA was calculated as before from voltage and current, and its inflation was controlled automatically using the syringe driver. Having demonstrated a suitable resistance–strain relationship (repeatable with an approximately one-to-one mapping between resistance and strain), tap water was used for all proprioceptive bending FFA experiments. Also, in all experiments with the proprioceptive bending FFA, the current sine wave used to determine resistance had an amplitude of 100 μA and frequency of 1000 Hz. As with the other proprioceptive FFA, video footage was captured and synchronized using an LED. To determine bending angle, two rectangular red markers were attached to each end of the FFA, and angle was calculated from video footage of the experiment using MATLAB image processing commands. For experiments with the proprioceptive bending FFA, the actuator was placed side down on a laboratory desk and filmed from above. There was some friction present between the actuator and the desk surface, however, the surface was not sticky and the actuator mass was low so frictional forces were kept low.

**FIG. 11.**
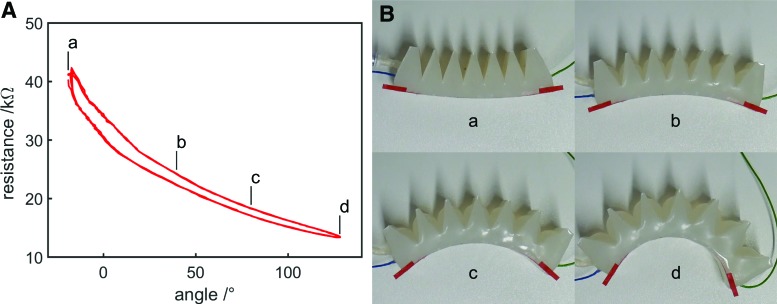
**(A)** Resistance variation with angle as the proprioceptive bending FFA was inflated and deflated three times automatically. **(B)** Video snapshots showing the curvature of the actuator during the experiment. Video snapshots labeled **a, b, c,** and **d** correspond to the regions **a, b, c,** and **d** in **(A)**. Color images available online at www.liebertpub.com/soro

Figure 11 shows results from inflation and deflation experiments with the proprioceptive bending FFA. Figure 11B shows frames from video footage of the experiment, with the labels a, b, c, and d corresponding to the regions a, b, c, and d in Figure 11A. A video of the experiment alongside live data is available in Supplementary Video S2. Resistance was reduced as the actuator was inflated and increased as the actuator was deflated. At point a, the applied pressure was lower than atmospheric pressure and so the actuator was deflated further than its rest position, resulting in deflection in the opposite direction to that of inflation. At this point, resistance was increased to a value greater than at-rest resistance (at a bending angle of 0°), demonstrating useful resistance variation when the actuator volume was increased (applied pressure greater than atmospheric pressure) as well as when reduced (applied pressure lower than atmospheric pressure) beyond its rest value.

**FIG. 12.**
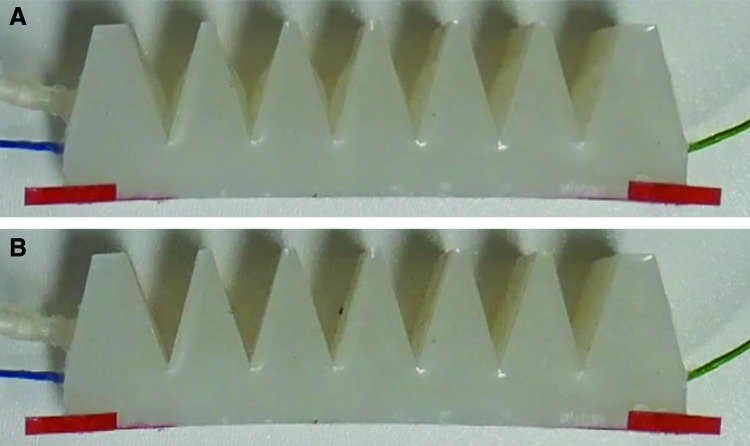
Video snapshots with the actuator at the same bending angle (6.4°), during **(A)** inflation and **(B)** deflation. The actuator walls bulge outward during inflation and buckle inward during deflation, resulting in a difference in cross-sectional area and therefore resistance. Color images available online at www.liebertpub.com/soro

Some hysteresis is visible, again attributed to the flexibility of the actuator walls, which bulge outward during inflation and buckle inward during deflation, resulting in a difference in cross-sectional area at a particular actuator bending angle, which influences resistance. This effect can be seen in Figure 12, which compares the actuator's appearance during inflation and deflation when at the same bending angle (around 6.4°, where hysteresis is large). Again, repeatability is good; Figure 11 shows three inflation and deflation cycles.

The inversely proportional resistance–inflation relationship shown in Figure 11 contrasts with that of the proprioceptive linear FFA, for which the resistance–inflation relationship was proportional (Fig. 9). This is attributed to the differing changes in geometry that occur when the two actuators are inflated. For the proprioceptive linear FFA, inflation mainly increased length while cross-sectional area was only slightly altered (until the actuator was overfilled). For the proprioceptive bending FFA, the inextensible mesh layer prevented the actuator length from changing significantly as the actuator was inflated. Conversely, the actuator bulged considerably when inflated, increasing its cross-sectional area. Since resistance is proportional to length and inversely proportional to cross-sectional area, overall resistance was reduced as the actuator was inflated. Measurement of resistance in this case is mostly measurement of cross-sectional area.

### Bandwidth

**FIG. 13.**
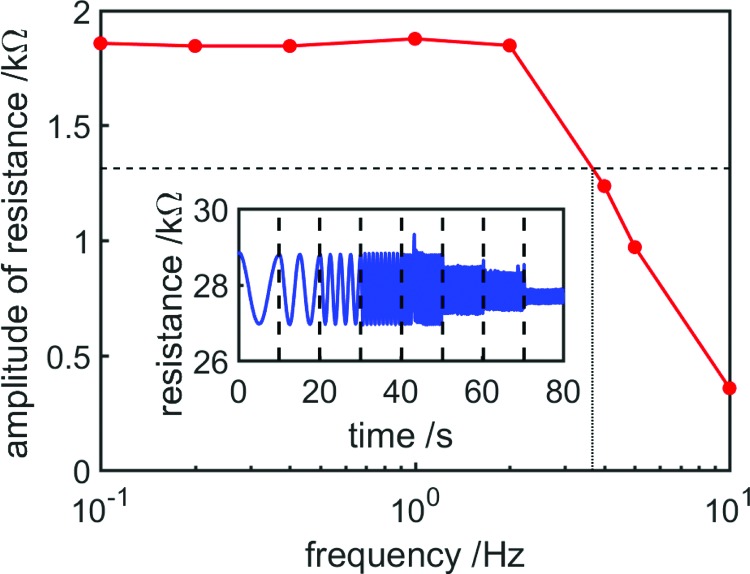
Bandwidth characterization of the proprioceptive bending FFA showing average resistance amplitude at driving frequencies ranging from 10^*−*1^ to 10 Hz. The *dashed line* shows the −3 dB cutoff, and the *dotted line* shows the interpolated value at which average resistance amplitude falls below the −3 dB cutoff. The sensor bandwidth is ∼3.665 Hz. The *inset* figure shows actual resistance variation during the 80-s experiment—*dashed lines* show transitions between oscillation frequencies. Color images available online at www.liebertpub.com/soro

An important characteristic of the proprioceptive FFAs proposed in this article is their bandwidth, which should be sufficiently high to allow them to sense strain at actuation frequencies suitable for useful tasks. An experiment to investigate the sensor bandwidth was performed, in which the syringe driver enforced a sinusoidal oscillation in volume (amplitude 1 mL) while resistance was recorded. The frequency of the oscillation was held constant for 10 s before being increased. Eight oscillation frequencies were used from 10^−1^ to 10 Hz. The sensor bandwidth (at the −3 dB cutoff) was ∼3.665 Hz (Fig. 13).

### Control

Having characterized resistance as a sensed variable to infer strain, we demonstrate its use as a feedback variable to control the proprioceptive bending actuator. First, a sensor model (an equation relating the sensed variable to the inferred variable) is required. Various sensor models will match the experimental data suitably well for control purposes, and the exact structure of the relationship will depend on Pouillet's Law and how the actuator geometry varies with the inferred variable. For example, if volume were the inferred variable, then for the cylinder of saltwater (Fig. 3), for which length varies linearly with volume and cross-sectional area is invariant, behavior is well matched by the equation

\begin{align*}
R = aV + b \tag{2}
\end{align*}

where *R* is resistance, *V* is volume, and *a* and *b* are constants, since resistance is proportional to length according to Pouillet's Law. In contrast, a cylindrical flexible actuator that mostly expands radially when inflated would be better matched by

\begin{align*}
R = \frac { a }  { V } + b , \tag { 3 } 
\end{align*}

Since according to Pouillet's Law, resistance is inversely proportional to cross-sectional area, and cross-sectional area is proportional to volume when length is conserved. Assuming resistivity is invariant and the FFA is not auxetic, these are the two extremes of possible sensor models; for most FFAs with simple geometries, length and cross-sectional area will both increase to some degree as the actuator is inflated, and the best sensor model will be some combination of Equations (2) and (3).

**FIG. 14.**
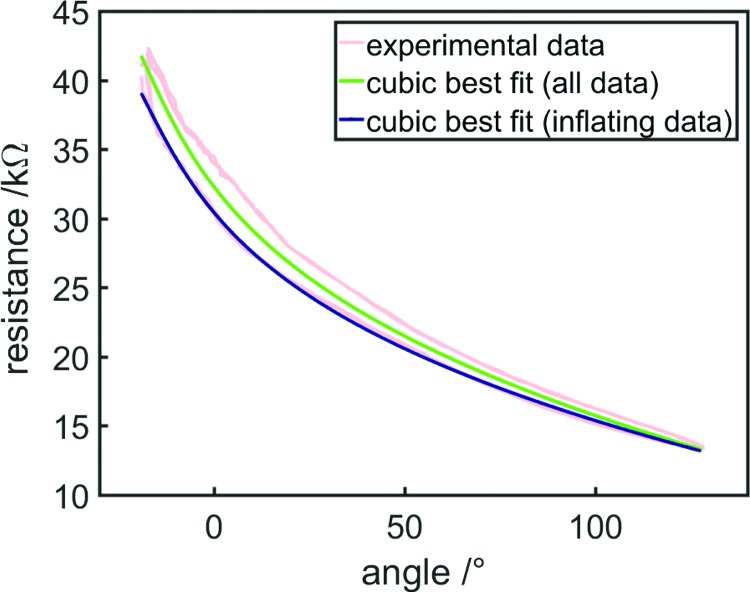
Experimental data relating resistance and bending angle for the proprioceptive FFA (*light red*), along with best fit third-order polynomials for all (*green*) and only inflating (*blue*) data. Color images available online at www.liebertpub.com/soro

For the purposes of control, it is only necessary that a sensor model should be have a one-to-one mapping between resistance and the sensed variable and should match the data well. To capture a simple equation that could be easily integrated into a controller, we chose to fit the data using a best fit (least-squares) third-order (cubic) polynomial, which had an R-squared value of 0.9213. Figure 14 shows the best fit polynomial (green) and experimental data (light red point cloud) from the bending FFA characterization (Fig. 11).

This best fit polynomial was used as a sensor model to estimate bending angle from resistance, and this inferred bending angle was used as the feedback variable in our controller. We used a simple proportional position controller, through which the difference between target and current (estimated) position was multiplied by a gain to determine the change in volume required. This change in volume was then enforced by the syringe driver, after which resistance was measured, and the cycle continued. Three position control tasks were attempted: a step function with amplitude 60°, a step function with amplitude 120°, and a two-period sinusoidal oscillation with amplitude 60°, centered at 60°.

**FIG. 15.**
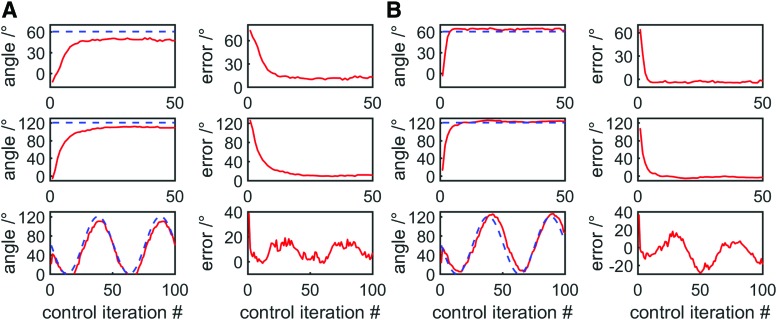
Position control tasks using resistance as a feedback variable, target bending angles are shown as *dotted blue* lines. From *top*: step function with an amplitude of 60°, step function with an amplitude of 120°, two periods of a 60° amplitude sinusoidal oscillation centered at 60°. The sensor model greatly influences results: **(A)** shows results using a cubic best fit of all resistance-length data, while **(B)** shows results using a cubic best fit of only inflating data. Color images available online at www.liebertpub.com/soro

Results can be seen in Figure 15A. Some steady-state error (∼10°) can be seen for the step function control tasks—this is because the best fit polynomial is centered within the hysteresis curve relating resistance and bending angle. Since resistance is lower when inflating compared with the best fit polynomial, and the actuator inflated to reach its target, this sensor model tended to overestimate the actuator's current bending angle.

To account for this, a second best fit polynomial was generated to be used as a second sensor model, using only a subset of data: the data from when the actuator was inflating. This second polynomial is shown in Figure 14. The same control tasks were performed using this polynomial to estimate bending angle, and steady-state error was greatly reduced for the step tasks, to a maximum of ∼5° (Fig. 15B). However, for the sinusoidal control task, in the sine wave's second quadrant (in which the actuator was being deflated), error was increased because the sensor model further *underestimated* bending angle compared with the previous best fit polynomial. Nonetheless, the largest steady-state error was still ∼20°. This error might be considered large for traditional rigid robotic systems, however, soft actuators may not require such precise control because their inherent compliance allows them to adapt to their environment and perform useful tasks despite control error.^1^

**FIG. 16.**
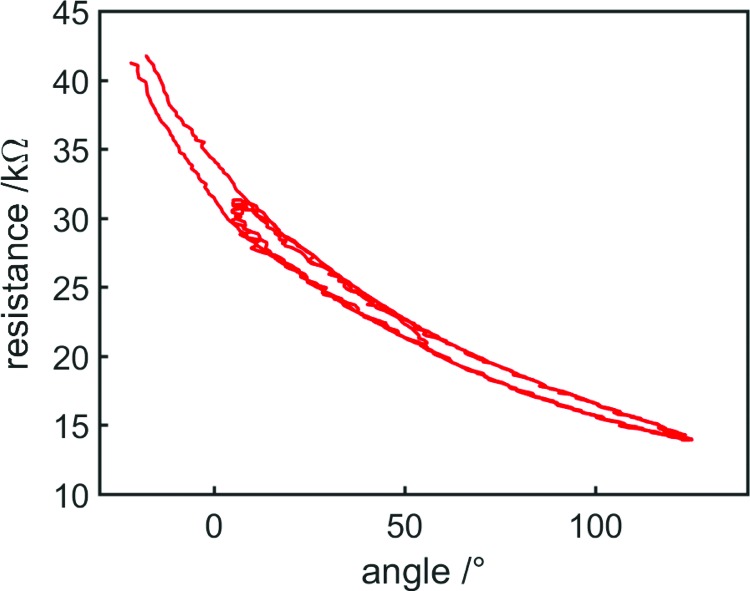
Resistance variation with bending angle during the sinusoidal control task using the second sensor model. As the sensor alternates between inflation and deflation, it returns to the upper or lower hysteresis curves depending on its state. Color images available online at www.liebertpub.com/soro

In the future, error can be reduced by using a more complex controller—here only a simple proportional controller was used, while a future controller can include derivative and integral components. In addition, steady-state error can be further reduced by improving the sensor model—it is known that the sensor exhibits hysteresis, and the sensor reliably returns to its upper and lower hysteresis curves when alternating between inflation and deflation (Fig. 16). As such, it should be possible to greatly reduce over- and underestimation of bending angle using a sensor model that includes the state of the actuator to account for hysteresis, using one relationship between resistance and angle when the actuator has just been inflated and another when it has just been deflated.

### Effect of temperature

**FIG. 17.**
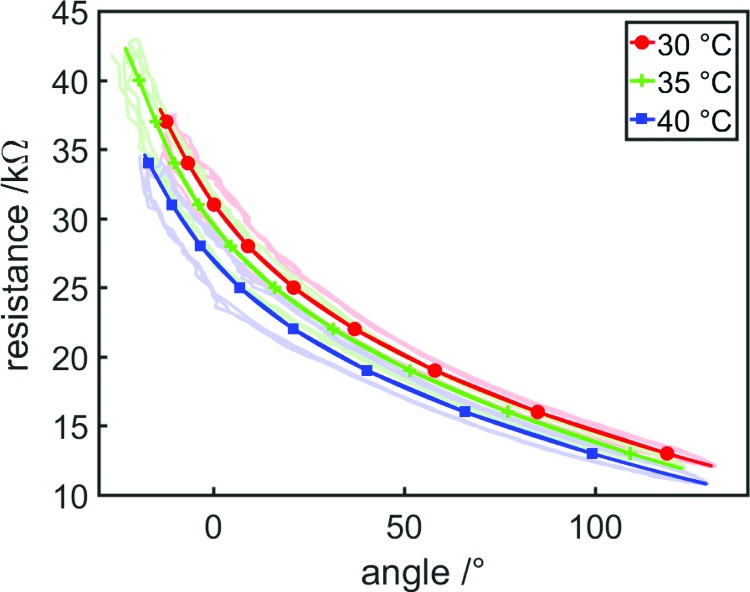
Resistance variation with bending angle at different tap water temperatures. Best fit (least squares) third-order polynomials are shown as *solid lines* (with markers to aid identification), and experimental data are shown as *lightly colored* point clouds. Resistivity was reduced at higher temperatures. Color images available online at www.liebertpub.com/soro

It is known that the conductivity of saltwater solutions varies with temperature; the variation can be as high as 2% per 1°C.^47^ The effect of tap water temperature on the resistance–angle relationship of the proprioceptive bending FFA was investigated. Tap water was controlled to an exact temperature using a hot plate with thermistor feedback (CD162; Stuart Equipment, England) before being pumped into the syringe driver and actuator. The syringe driver then inflated and deflated the bending FFA three times and resistance was calculated as before. Figure 17 shows resistance variation with angle at different tap water temperatures. At bending angles of 0° resistance, values are 31.1, 29.5, and 26.9 kΩ for temperatures of 30, 35, and 40°C. Resistance at a bending angle of 0° was reduced by 5.14% as temperature was increased from 30°C to 35°C and by 13.50% as temperature was increased from 30°C to 40°C.

If temperature variation is likely to be high, this resistance variation could cause erroneous strain estimates when using resistance to infer strain, and should be accounted for—one option is the inclusion of a temperature sensor within the actuator, which measures the fluid temperature and adjusts the equation relating resistance and strain accordingly. All other experiments in this article were performed in a 20°C temperature-controlled room and the conductive fluids were stored at room temperature before use to prevent temperature variation from influencing results.

### Constraint detection

Previous work has used fluid pressure as a feedback variable to infer strain, in the same way as strain is inferred from resistance in this article.^41,42^ In these previous works, and in the experiments presented thus far in this article, the actuators are free to move and unconstrained. When the FFA is free to move, inferring strain from pressure or resistance is possible, because there is a mapping between steady-state actuator volume, actuator strain, internal pressure, and resistance (although hysteresis should be accounted for). However, if a constraint or obstacle blocks the path of the FFA, this is no longer the case, as variables will depend on the external load. Some detection of constraints is necessary for a proprioceptive actuator, since generally for useful tasks, actuators should apply loads to their environments, and so, they will inevitably experience loads as well.

**FIG. 18.**
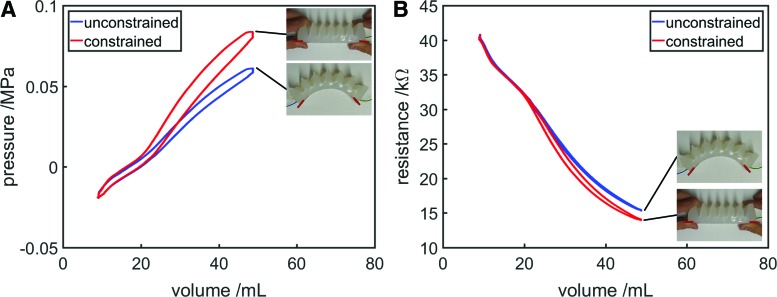
Variation in **(A)** fluid pressure and **(B)** resistance for the proprioceptive bending FFA as volume is controlled, comparing a free-to-move state (*blue*) with one where a constraint prevents bending (*red*). Color images available online at www.liebertpub.com/soro

We performed an experiment to determine whether it would be possible to detect the presence of a constraint by examining the resistance–volume relationship of the proprioceptive bending FFA. For this experiment, the syringe driver inflated and deflated the actuator once, and then, a constraint was applied to the actuator (it was held straight and prevented from bending). The syringe driver then inflated and deflated the actuator once more. Volume, pressure, and resistance were recorded throughout the entire experiment. Results are shown in Figure 18.

It was possible to detect that the system was constrained in some way by examining either the pressure–volume or the resistance–volume relationship of the actuator. Flexible fluidic systems tend to inflate in a way that minimizes the material stresses, however, if constrained in some way, greater local strains (and therefore stresses) develop in unconstrained areas, resulting in a higher overall pressure at a given volume (Fig. 18A). Also, if the actuator is constrained in some way, an increase in fluid volume results in an increase either in the actuator's area or length, depending on the nature of the constraint, compared with its unconstrained shape. In either case, resistance will be lower or higher than the unconstrained resistance at that volume (Fig. 18B).

In addition to simply detecting a constraint, as is possible by measuring pressure and volume, measurement of resistance and volume allows something about the nature of the constraint to be determined. Since resistance is proportional to length and inversely proportional to area, it contains geometric information about the aspect ratio of the FFA: an increase in resistance implies that actuator has become longer or thinner, while a reduction in resistance implies the actuator has become shorter or wider. As such, by observing the direction of resistance variation at a given volume compared with an unconstrained state, it can be determined what effect the constraint has had on the FFA geometry. In the future, it should be possible to use the amount by which the resistance–volume and pressure–volume relationships have deviated from their unconstrained paths to infer how constrained the actuator is, which could be used to predict the strain of the actuator even when under load, and to characterize obstacles that the actuator is interacting with.

## Discussion and Conclusion

This article introduces the concept of using a conductive liquid to perform both the functions of a proprioceptive (self-sensing) soft actuator. The working fluid drives actuator deformation and simultaneously acts as the strain-sensing component for detecting actuator deformation. Since the fluid is conductive, and the length and cross-sectional area of fluid contained within the actuator vary as it is inflated or deflated, measurement of resistance using embedded electrodes allows the actuator to act as a sensor. This concept is advantageous over the traditional method of combining a standalone soft actuator and standalone soft sensor because it reduces the actuator-sensor system volume footprint and mass and can reduce complexity. Liquid working fluids have several advantages over gaseous ones, such as lower volume requirements because of lower compressibility, greater force transmission, and now, resistive strain-sensing capabilities, however, their downsides include lower backdrivability and potential risks of damage caused by leaking.

The concept was examined in two test cases: a cylinder of saltwater with circular electrodes of diameters close to that of the cylinder, and a rectangular bath of saltwater with small electrodes. Generally, the saltwater behaved like an ideal conductor according to Pouillet's Law, although the behavior is more complex near the electrodes and this effect can cause nonlinear behavior if the electrodes are close. The conductive fluid proprioception concept was then used to construct two proprioceptive FFAs, a linear actuator and a bending actuator. In both cases, resistance was shown to be a good predictor of the actuator's strain, although some hysteresis was present.

The bandwidth of resistance variation as a sensing variable to infer length was tested and found to be ∼3.665 Hz, which is suitable for many soft robotic tasks, for example, for position control in robotic surgery. We then demonstrated how resistance can be used in a control loop, by using resistance to estimate bending angle, which was used as a feedback variable. The proprioceptive bending FFA was controlled to respond to a step input and sinusoidal target function. Maximum error was around 20°, which, although high for traditional hard robotic systems, is sufficient for some soft robotic tasks because of the flexibility of soft robotic actuators.^1^ We examined the effect of temperature on resistance–strain behavior and discussed how it could be addressed. Finally, we showed how resistance–volume behavior can be used to detect when the actuator is constrained, and also provide information on how the constraint has changed the actuator's geometry compared to its unconstrained shape. While initially demonstrating proprioception using saltwater as a conductive working fluid, we showed that tap water contains sufficient trace salts to be useful as a conductive strain-sensing fluid, and demonstrated subsequent experiments using tap water. This demonstrates the ready adoptability of the conductive fluid proprioception concept: all current FFAs that already use water as the working fluid could be adapted to be proprioceptive simply through the addition of electrodes. Alternatively, for FFAs that have fluid ports at both ends, electrodes need not be added to the actuator at all; sensing of the port to port resistance would provide an estimate of actuator strain.

In this article, only two electrodes were used for each actuator; however, multiple electrodes could be included along the length, height, and depth of an FFA allowing for local sensing of strain and multiple degree of freedom proprioception.

Hysteresis is present in all FFAs (including the proprioceptive ones demonstrated here) and is caused by the inherent flexibility of these actuators coupled with their geometry. Depending on whether the actuator is being inflated or deflated, the actuator walls may bulge outward (due to higher pressure) or buckle inward (due to lower pressure). This alters the actuator cross-sectional area and thus resistance. To account for this, proprioceptive FFAs should be characterized both during inflation and deflation, and the state of the actuator (inflating or deflating) be included in the sensor model.

For the proprioceptive linear FFA, resistance increased as the actuator was inflated, whereas for the proprioceptive bending FFA, the resistance–inflation relationship was inversely proportional. This is attributed to the differing geometric variation as these actuators were inflated. For the proprioceptive linear FFA, inflation mostly increased length and cross-sectional area was not changed much, whereas for the proprioceptive bending FFA, inflation only slightly increased length but increased the cross-sectional area considerably. Since resistance is proportional to length and inversely proportional to cross-sectional area, the resistance–inflation relationship of the two systems differed. This behavior highlights the interplay of the effect of pressure-induced lengthening and pressure-induced thickening on resistance for proprioceptive FFAs.

As discussed in the Materials and Methods section, generally voltages were kept lower than the relevant oxidation and reduction potentials of the saltwater. In experiments not presented here, where high-amplitude current sine waves were applied, potentials were high enough for electrolysis to occur. This was a problem not only because it affected the conductive liquid (aqueous sodium chloride reacts to poisonous chlorine, explosive hydrogen, and corrosive sodium hydroxide) but also because the oxidation and reduction reactions caused degradation of the electrodes. When choosing excitation voltages or currents, it is important that care be taken to keep voltages sufficiently low.

Typically, the frequency of the current sine wave used to determine resistance was 1000 Hz, however, we have shown that even at 10 Hz, resistance could be used as a predictor of actuation strain. The exact frequency necessary for a good resistance–strain relationship will depend on the frequency of strain variation—in general, the excitation frequency should be considerably larger than the frequency of strain variation to ensure high accuracy and must certainly be above the Nyquist frequency.

In these experiments, a laboratory galvanostat was used to determine resistance. A lower cost alternative could involve a cheap microcontroller generating a controlled voltage sine wave and measuring resistance with a potential divider arrangement. It is therefore possible to fabricate small, low-cost proprioceptive FFAs using readily available and inexpensive components.

An additional advantage of the use of saltwater and tap water within soft robotic components is biocompatibility.^48^ The silicone rubber used to fabricate the actuators described in this article is nontoxic, tap water is safe for consumption, and saltwater is biocompatible (commonly used medical “normal” saline has ratios of salt to water 1:111 and “quarter-normal” saline is 1:453). The electrodes used in the bending FFA were gold plated and also biocompatible. Thus, such proprioceptive actuators could be included in bionic healthcare components such as orthotics, prosthetics, or even replacement muscles allowing both actuation and sensing in a biocompatible device.

## Supplementary Material

Supplemental data

Supplemental data

Supplemental data

## Data Access Statement

Data necessary to support the conclusions are included in the article.
