# Supplementary material for: Proprioceptive Flexible Fluidic Actuators Using Conductive Working Fluids
Source: Soft Robot. 2018 Apr 1;5(2):175–89. doi: 10.1089/soro.2017.0012 (PMC5905876; doi:10.1089/soro.2017.0012)
Supplement: Supplemental data [file Supp_Video1.zip › Supp_Video1.pdf]

Supplementary Data

SUPPLEMENTARY VIDEO S1. A proprioceptive linear flexible fluidic actuator. A rubber bellow was adapted as a linear actuator. Electrode structures comprising acrylic disks with outer holes to allow throughflow of liquid and a central copper conducting electrode were added at each end of the rubber bellow. The bellow was filled and actuated with tap water, which is conductive due to the presence of trace salts. A 1 mA amplitude, 1000 Hz frequency current sine wave was maintained using a galvanostat and applied voltage was recorded. Current and voltage were used to calculate resistance. Actuator length was recorded using a laser displacement meter.
