# Supplementary material for: Proprioceptive Flexible Fluidic Actuators Using Conductive Working Fluids
Source: Soft Robot. 2018 Apr 1;5(2):175–89. doi: 10.1089/soro.2017.0012 (PMC5905876; doi:10.1089/soro.2017.0012)
Supplement: Supplemental data [file Supp_Video2.zip › Supp_Video2.pdf]

SUPPLEMENTARY VIDEO S2. A proprioceptive bending flexible fluidic actuator. A bending flexible fluidic actuator was fabricated using a two-part casting process from silicone rubber and an inextensible mesh layer. Gold-plated 1 mm diameter test plugs were added at each end of the actuator as electrodes, produting 7 mm into the actuator's internal chamber. The actuator was filled and actuated with tap water, which is conductive due to the presence of trace salts. A 100 μA amplitude, 1000 Hz frequency current sine wave was maintained using a galvanostat and applied voltage was recorded. Current and voltage were used to calculate resistance. Bending angle was calculated from video footage of the experiment using MATLAB image processing commands.
